# Vitamin D status in overweight and obese Malaysian school children and its relationship with metabolic syndrome

**DOI:** 10.1186/1687-9856-2015-S1-O50

**Published:** 2015-04-28

**Authors:** Nurshadia Samingan, Azriyanti Anuar Zaini, Fatimah Harun, Nor Asiah Muhammad, Normi Mustapha, Muhammad Yazid Jalaludin

**Affiliations:** 1 Department of Paediatrics, University Malaya, Kuala Lumpur, Malaysia; 2 Institute of Medical Research, Kuala Lumpur, Malaysia; 3 Department of Research Methodology and Biostatics, University Sains Malaysia, Kubang Kerian, Kelantan, Malaysia

## 

Obesity is a rising health problem, with increasing prevalence in children and adolescents. Lower vitamin D is linked to increased adiposity and higher risk of metabolic syndrome. However, evidence from tropical Asian countries is limited, especially in children and adolescents.

To examine the relationship between vitamin D level and BMI, abnormal glucose profile, insulin resistance and metabolic syndrome markers in the overweight/ obese secondary school children.

A cross sectional study in multiethnic secondary school children aged 13 -17 years was performed. Anthropometric measurements: height, weight, waist circumference and blood pressure were obtained. Blood for fasting glucose/ lipids/ insulin, and vitamin D (25 (OH)D) were taken. Oral glucose tolerance test was also performed. Insulin resistance indices were calculated based on homeostasis model assessment (HOMA) index.

A total of 543 subjects were enrolled. Forty eight percent were overweight/obese. Most of them (62%) were vitamin D deficient (<50 nmol/L), 32% were vitamin D insufficient (50 to <75 nmol/L) and only 6% were vitamin D sufficient (≥ 75 nmol/L). Mean 25(OH)D in the overweight/ obese group was 44.3±15.9nmol/L and was significantly lower compared to the non overweight/obese group (47.9±19.4nmol/L); (p=0.018). Females had lower mean vitamin D level (43.2±15.9nmol/L) compared to males (53.6±19.6nmol/L) (p<0.001). The Chinese had the highest mean vitamin D level (65.9±16.4nmol/L), followed by Malays (44.2±16.9nmol/L) and Indians (39.5±13.3nmol/L) (p<0.001).

Among those who were overweight and obese, metabolic syndrome was present in 58 (22%) of them. No significant relationship was found between 25(OH)D level and abnormal glucose profile, insulin resistance and metabolic syndrome markers among the overweight/ obese participants. The overweight/ obese females had 78% prevalence of vitamin D deficiency, compared to 59% in overweight/ obese males. Overweight/ obese Indians had the highest prevalence of vitamin D deficiency (82%) followed by Malays (70%) and Chinese (20%).

Vitamin D deficiency is highly prevalent in Malaysian adolescents despite an abundance of sunlight. Lower vitamin D levels are associated with female gender, ethnic groups with darker skin and obesity. However, no relationship was found between vitamin D deficiency and metabolic syndrome.

